# Host interactions of *Lactococcus lactis* and *Streptococcus thermophilus* support their adaptation to the human gut microbiota

**DOI:** 10.1128/aem.01547-25

**Published:** 2025-11-04

**Authors:** Gabriele Andrea Lugli, Chiara Argentini, Chiara Tarracchini, Giulia Longhi, Leonardo Mancabelli, Massimiliano G. Bianchi, Giuseppe Taurino, Alberto Amaretti, Francesco Candeliere, Ovidio Bussolati, Christian Milani, Francesca Turroni, Marco Ventura

**Affiliations:** 1 Laboratory of Probiogenomics, Department of Chemistry, Life Sciences, and Environmental Sustainability, University of Parma 9370, Parma, Italy; 2 Microbiome Research Hub, University of Parma 9370, Parma, Italy; 3 Department of Medicine and Surgery, University of Parma 9370, Parma, Italy; 4 Laboratory of General Pathology, Department of Medicine and Surgery, University of Parma 9370, Parma, Italy; 5 Department of Life Sciences, University of Modena and Reggio Emilia 9306, Modena, Italy; 6 Biogest-Siteia, University of Modena and Reggio Emilia 9306, Reggio Emilia, Italy; Norwegian University of Life Sciences, Ås, Norway

**Keywords:** lactic acid bacteria, genomics, metagenomics, microbiome, host-microbe interaction

## Abstract

Within the human gut microbiota, lactic acid bacteria (LAB) play a crucial role in host health by producing lactic acid, which has been shown to shape microbial interactions and support intestinal homeostasis. However, despite their importance, there are limited insights regarding how LAB species interact with the host and other gut commensals. In this study, the investigation of the human gut microbiota of 10,000 healthy adults allowed the identification of *Lactococcus lactis* and *Streptococcus thermophilus* as commonly detected food bacteria. Further *in silico* analyses led to the identification of reference strains of the *L. lactis* and *S. thermophilus* species within the human gut, represented by PRL2024 and PRL2025 strains, respectively, which can represent nomadic bacteria. *In vitro* experiments revealed that both strains are ecologically adapted to survive and interact within the human gastrointestinal tract, while also highlighting their metabolic capacity to utilize a broad range of carbon sources. Specifically, the lactose metabolism was investigated, revealing that *S. thermophilus* PRL2025, despite high lactic acid output, incompletely metabolizes galactose, whereas *L. lactis* PRL2024 ensures full galactose utilization with lower acid production.

**IMPORTANCE:**

The identification and functional characterization of *Lactococcus lactis* PRL2024 and *Streptococcus thermophilus* PRL2025 as human-adapted reference strains provide a valuable foundation for further in vivo experimentation. Given their ecological resilience, metabolic versatility, and interaction potential with beneficial gut microbes, these strains represent promising candidates as microbiota-targeted functional foods.

## INTRODUCTION

Lactic acid bacteria (LAB) are a group of gram-positive, non-spore-forming, typically rod- or cocci-shaped bacteria producing lactic acid as a major metabolic end product of carbohydrate fermentation (1). They are facultative anaerobes or microaerophilic and are commonly found in environments rich in carbohydrates, such as milk, plant materials, and the gastrointestinal tracts (GITs) of animals (2, 3). LAB play a crucial role in the production of fermented foods and beverages, including yogurt, cheese, sauerkraut, kimchi, and pickles. They are also important probiotics, known for their potential health benefits, including improving gut health and supporting the immune system (4). Common genera of lactic acid bacteria include *Lactobacillus* (currently reclassified into 25 different genera), *Lactococcus*, *Leuconostoc*, *Pediococcus*, and *Streptococcus* (5, 6). Among them, *Lactococcus lactis* as well as *Streptococcus thermophilus* and various *Lactobacillus* species (reclassified as *Lacticaseibacillus*, *Lactiplantibacillus*, *Latilactobacillus*, *Levilactobacillus*, *Fructilactobacillus*, and *Companilactobacillus* genera) are commonly found in fermented foods (7–9). These food-borne microorganisms play a crucial role in fermentation, enhancing the shelf life, sensory profile, and nutritional value of these foods.

LAB produce various metabolites, such as organic acids, bacteriocins, amino acids, exopolysaccharides (EPSs), and vitamins, which play a key role in regulating gut microbiota and promoting host health (10, 11). These metabolites help maintain gut welfare, support intestinal homeostasis, and enhance resistance to pathogens. Among these metabolites, LAB produce lactic acid as a major end product of carbohydrate fermentation via two main pathways, i.e., homofermentative and heterofermentative (12). The production of lactic acid acidifies the surrounding environment, often dropping the pH to levels as low as 4.0 or lower. This is an effective strategy for LAB to inhibit competing microorganisms, including pathogenic bacteria (13, 14). However, while lactic acid generally inhibits many microbes, some bacteria thrive in acidic environments established by LAB, such as acidophilic bacteria (15). Within the gut microbiota, *Veillonella parvula* is a commensal anaerobic bacterium known for its ability to utilize lactic acid as a primary carbon source, converting it into propionate and acetate (16). Other intestinal microorganisms, including *Anaerobutyricum hallii* and *Clostridium butyricum*, utilize lactic acid to produce butyrate, demonstrating their potential to mitigate various human pathologies associated with gut health and inflammation (17). Furthermore, lactic acid production by LAB can establish favorable conditions for other beneficial bacteria, such as members of the genus *Bifidobacterium* (18).

LAB, such as *S. thermophilus* and *L. lactis*, which are widely used in dairy fermentation and commonly found in fermented foods, are transient members of the human gut microbiota, yet they can modulate its composition and metabolism and exert effects on host health (19, 20). Previous metagenomic screening of the human gut microbiota in association with food metagenomes revealed that LAB are typically present in low abundance in the human gut, with their prevalence influenced by age, lifestyle, and geography (21, 22). Additionally, comparative genomics has revealed that closely related LAB strains are found in both food and gut environments, suggesting that fermented foods represent a significant source of nomadic LAB in the human gut metagenome (22).

In this study, we investigate the microbial biodiversity of the gut microbiota in 10,000 healthy adults, identifying the most prevalent LAB within the population. Then, we applied a recent bioinformatics pipeline to classify reference strains for various species associated with the human gut microbiota (23), leading to the identification of two representative strains, *L. lactis* PRL2024 and *S. thermophilus* PRL2025, which were isolated from food matrices and evaluated for their potential to interact with the human gut environment. Through *in vitro* models (23), the genetic and ecological features of these reference strains were investigated to dissect their capability to interact with the host.

## RESULTS AND DISCUSSION

### Identification of LAB reference strains within the human gut microbiota

To identify which LAB species were the most prevalent in the gut microbiota of healthy adults, 10,000 shotgun metagenomic samples were explored (23). The most prevalent human gut microbiota LAB species of food origin were *S. thermophilus* (prevalence of 16.3%) and *L. lactis* (prevalence of 4.6%), followed by *Enterococcus faecium*, *Weissella cibaria*, *Lactobacillus paracasei*, and *Lactobacillus delbrueckii* with a prevalence between 2.5% and 1.0% (Table S1). Furthermore, three additional *Lactobacillus* species of non-food origin were identified, i.e., *Lactobacillus ruminis*, *Lactobacillus salivarius*, and *Lactobacillus mucosae*, with a prevalence ranging from 6.5% to 1.6% (Table S1). The data collected were consistent with a previous large-scale screening of gut metagenome samples, which identified *S. thermophilus* and *L. lactis* as the most prevalent LAB species in the human gut (21), as well as the most represented species whose genomes were recovered from metagenomic samples of dairy products (22). Scientific evidence suggests that these food-related bacteria are transient within the gut microbiota (19, 20). In this context, a longitudinal *in silico* screening was performed on 35 subjects whose gut metagenomes were sampled over seven consecutive days at baseline (pre-treatment) (24). Interestingly, *S. thermophilus* showed stable colonization (prevalence >85% across samples) in seven individuals, whereas *L. lactis* was observed to be stable in only four subjects (Fig. S1; Table S2). In this regard, it is worth noting that scientific evidence also supports the hypothesis that members of the food microbiota can reach and colonize the human gut via horizontal transmission through food ingestion (25–27).

Based on collected data, isolation efforts were placed to cultivate strains of the two most prevalent LAB species of food origin from human stool samples and different food matrices (Table 1), including various cheeses, processed meats, and fermented vegetables (28). Among isolated strains, 7 were identified as belonging to *L. lactis* and 19 to *S. thermophilus*, and the chromosomes of these strains were subjected to whole-genome sequencing to have a glimpse of their genomic content (Table 1).

**TABLE 1:** General genome features of 26 strains of *L. lactis* and *S. thermophilus*

Strain name	Species	16S rRNA gene identity^*a*^	ANI screening^*a*^	Genome completeness	Genome contamination	Avg coverage	No. of contigs	Genome length	No. of genes	No. of rRNA genes	No. of tRNA genes	Origin
17C	*L. lactis*	100	97.7	100	0.32	152	103	2,517,471	2,483	7	55	Pecorino toscano
18C	*S. thermophilus*	100	99.3	99.89	0.32	125.7	29	1,813,995	1,905	4	45	Taleggio DOP
19C (PRL2025)	*S. thermophilus*	100	99.1	99.82	0.15	315.6	1	1,865,679	1,949	18	68	Toma Piemontese DOP
25C (PRL2024)	*L. lactis*	99.9	97.8	100	0.66	451.4	5	2,795,423	2,839	19	70	Stelvio DOP
26C	*S. thermophilus*	100	98.9	99.89	0.15	161.3	36	1,756,755	1,885	5	44	Bra DOP
27C	*S. thermophilus*	100	98.4	99.89	0.15	79.5	103	1,930,325	2,048	3	41	Caciocavallo DOP
50C	*S. thermophilus*	100	98.4	99.89	0.15	42.2	142	1,907,956	2,031	3	40	Caciocavallo DOP
51C	*S. thermophilus*	99.9	98.9	99.39	0.15	156.8	56	1,780,217	1,916	4	43	Siero latte
52C	*S. thermophilus*	100	98.4	99.89	0.19	125.2	116	1,899,834	2,019	3	41	Caciocavallo DOP
53C	*S. thermophilus*	99.9	98.9	99.83	0.15	123.6	59	1,747,866	1,876	4	44	Siero latte
54C	*S. thermophilus*	100	98.2	99.24	0.15	44.2	181	1,941,364	2,054	4	42	Caciocavallo DOP
55C	*S. thermophilus*	100	98.2	99.75	0.15	63.9	142	1,905,890	2,024	4	41	Caciocavallo DOP
56C	*S. thermophilus*	100	99.2	99.89	0.36	55.7	102	1,774,023	1,867	5	43	Montasio DOP
57C	*S. thermophilus*	99.9	98.8	99.83	0.15	112.3	53	1,785,715	1,921	5	43	Siero latte
58C	*S. thermophilus*	99.9	98.8	99.83	0.15	81.2	69	1,776,409	1,908	5	43	Siero latte
59C	*S. thermophilus*	99.9	98.8	99.83	0.15	107.2	67	1,737,495	1,870	5	44	Siero latte
60C	*S. thermophilus*	99.9	98.9	99.39	0.21	66	70	1,764,706	1,894	4	43	Siero latte
63C	*L. lactis*	100	98	100	0.95	340.2	67	2,525,339	2,531	7	57	Asiago DOP
65C	*L. lactis*	100	98.1	100	0.19	304.6	38	2,603,234	2,577	7	56	Murazzano DOP
66C	*S. thermophilus*	99.9	99.3	99.89	0.15	104.4	46	1,796,733	1,902	4	47	Murazzano DOP
68C	*S. thermophilus*	100	99.1	99.89	0.15	99.4	53	1,792,703	1,880	5	44	Montasio DOP
69C	*S. thermophilus*	99.9	98.8	99.39	0.15	89.5	54	1,766,982	1,901	5	43	Siero latte
71C	*S. thermophilus*	99.9	98.9	99.83	0.15	80.5	58	1,783,270	1,917	5	37	Siero latte
F01	*L. lactis*	100	98.1	100	0.95	315.4	57	2,484,030	2,489	7	56	Verza
F03	*L. lactis*	100	98.1	100	0.57	349.1	47	2,437,697	2,443	7	56	Cavolo cappuccio
FO9	*L. lactis*	100	98	100	0.95	92.7	62	2,482,314	2,490	7	56	Verza

^*a*^ *L. lactis* 14B4 (GCF_003176835.1) and *S. thermophilus* 4078 (GCF_020844005.1) used as reference.

The availability of the genetic information of each isolate allowed us to apply the Local Alternative Optimal Representative Strain (LAORS) pipeline (23), aiming to select reference strains for *in vitro* characterization. As previously applied for the identification of reference strains belonging to the genus *Bifidobacterium* (29–32), *L. lactis* and *S. thermophilus* strains were evaluated from a genetic, functional, and ecological perspective, allowing the selection of *L. lactis* PRL2024 (LAORS score of 8,672.2) and *S. thermophilus* PRL2025 (LAORS score of 8,753.76; Table S3). Therefore, the two selected strains were employed for long-read sequencing, allowing the reconstruction of the complete genome sequence of both isolates (Table 1) so as to correlate the genetic information with the subsequent *in vitro* evaluation of the biological features of the microorganisms.

### Insights into the carbohydrate metabolism of *L. lactis* PRL2024 and *S. thermophilus* PRL2025

**Fig 1:**
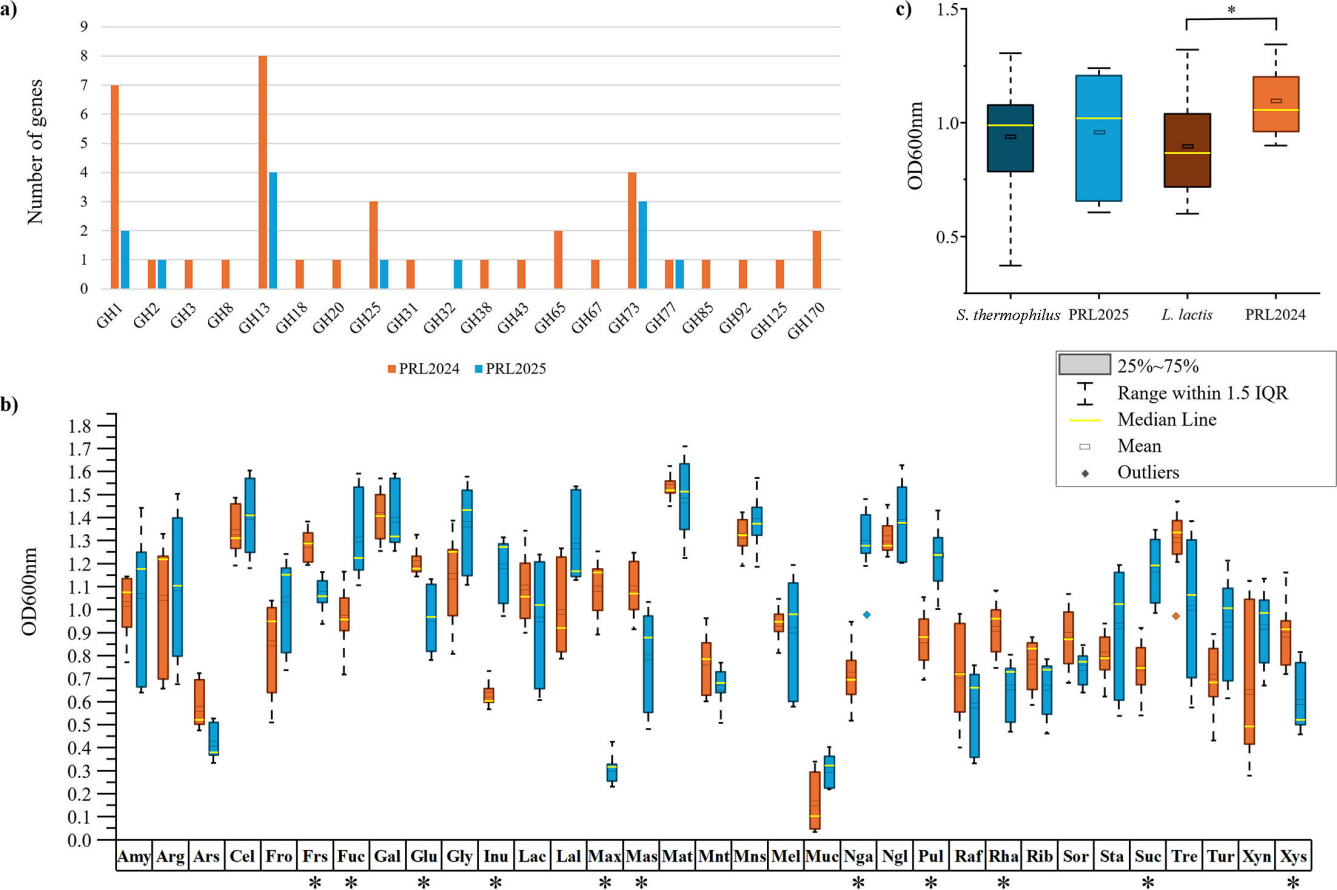
*In silico* and *in vitro* carbohydrate profiling of *L. lactis* PRL2024 and *S. thermophilus* PRL2025. Panel (**a**) shows the GH gene distribution in PRL2024 and PRL2025. Panel (**b**) exhibits the growth performance of both strains on different carbohydrates as measured by optical density at 600 nm (OD600nm). The following abbreviations are used for sugars: amylopectin (Amy), arabinogalactan (Arg), arabinose (Ars), cellobiose (Cel), fructooligosaccharides (Fro), fructose (Frs), fucose (Fuc), galactose (Gal), glucose (Glu), glycogen (Gly), inulin (Inu), lactose (Lac), lactulose (Lal), maltodextrin (Max), maltose (Mas), maltotriose (Mat), mannitol (Mnt), mannose (Mns), melibiose (Mel), mucin (Muc), N-acetyl-D-galactosamine (Nga), N-acetyl-D-glucosamine (Ngl), pullulan (Pul), raffinose (Raf), rhamnose (Rha), ribose (Rib), sorbitol (Sor), starch (Sta), sucrose (Suc), trehalose (Tre), turanose (Tur), xylan (Xyn), and xylose (Xys). Statistically significant values are indicated with an asterisk (Mann–Whitney test *P*-value < 0.05). Panel (**c**) displays a growth comparison of lactose between the two reference strains with respect to the other LAB isolates in the frame of this work (Table 1).

An *in silico* screening of the metabolic capabilities of the selected two LAB strains revealed 39 genes identified as glycosyl hydrolases (GHs), encompassing 19 different families in the genome of *L. lactis* PRL2024, and 13 genes in the chromosome of *S. thermophilus* PRL2025, distributed among seven GH families (Fig. 1a). In the glycobiome of PRL2024, most of the identified GHs were predicted to encode members of the GH13 family (eight genes), predicted to encode α-glucosidases, as well as seven genes encoding β-glucosidases of the GH1 family. Nevertheless, the remaining 24 predicted GHs, retrieved from the analysis, pointed to a broad usage of multiple carbon sources for this strain. This group includes α-mannosidases (GH38 and GH92), a β-galactosidase (GH2), a β-N-acetylhexosaminidase (GH20), an α-glucuronidase (GH67), an α-xylosidase (GH31), a 4-α-glucanotransferase (GH77), a β-N-acetylglucosaminidase (GH85), and a GH65 predicted to be involved in trehalose metabolism. Excluding genes predicted to encode for GH25 and GH73, which are usually associated with the biosynthesis of peptidoglycan, the predicted glycobiome of PRL2025 displayed α-amylases (GH13), β-glucosidases (GH1), a β-galactosidase (GH2), a GH32 foreseen to metabolize sucrose, and a 4-α-glucanotransferase (GH77).

The predicted metabolic capabilities of PRL2024 and PRL2025 were then investigated through *in vitro* assays using 33 carbon sources (Fig. 1b). Growth experiments were performed by using a de Man-Rogosa-Sharpe (MRS) medium without any carbon source, supplemented with one of 33 different carbohydrates (Fig. 1b), consisting of either plant- or host-derived glycans, which are commonly present in the adult human gut (33). Despite the different amount of GH families between PRL2024 and PRL2025 (Fig. 1a), strains displayed sizable growth performance (OD600 ≥ 1) on 14 and 20 commonly retrieved human gut carbohydrates, respectively (Fig. 1b). Both strains showed a good growth performance (OD600 ≥ 1) on amylopectin, arabinogalactan, cellobiose, fructose, galactose, glycogen, lactose, maltotriose, mannose, N-acetyl-D-glucosamine, and trehalose. In contrast, PRL2025 demonstrated high growth performances on nine sugars (Mann–Whitney test *P*-value < 0.01; Fig. 1b), i.e., fructooligosaccharides, fucose, inulin, lactulose, N-acetyl-D-galactosamine, pullulan, starch, sucrose, and turanose, while PRL2024 revealed an enhanced growth capability on glucose, maltodextrin, and maltose (Fig. 1b).

Results suggested that both strains are metabolically equipped to utilize a broader range of carbon sources. Nevertheless, *S. thermophilus* PRL2025 was found to be more prevalent in the human gut microbiota than *L. lactis* PRL2024 (Table S1). Our hypothesis is that the enhanced ability of *S. thermophilus* to colonize the human gut is due to its ability to interact with other microorganisms, thus taking advantage of the resulting trophic interactions. Interestingly, both reference strains demonstrated the ability to utilize lactose, which is a sugar commonly used by LAB in dairy fermentations. In this context, *L. lactis* and *S. thermophilus* are LAB widely employed in the food industry due to their capacity to metabolize lactose (34, 35). Therefore, their ability to convert lactose into lactic acid was investigated, focusing on the activity of their predicted β-galactosidase enzymes (GH2 family, Fig. 1a). To better understand the growth capability of the two reference LAB, the growth for all isolated strains on lactose was also evaluated (Table 1). While PRL2025 displayed a growth capability comparable with that of the other *S. thermophilus* isolated in the frame of this study (median OD600 of 1.02 and 0.99, respectively, Fig. 1c), PRL2024 showed a remarkably higher growth performance than the other *L. lactis* isolates (Mann–Whitney test *P*-value < 0.01; Fig. 1c).

**Fig 2:**
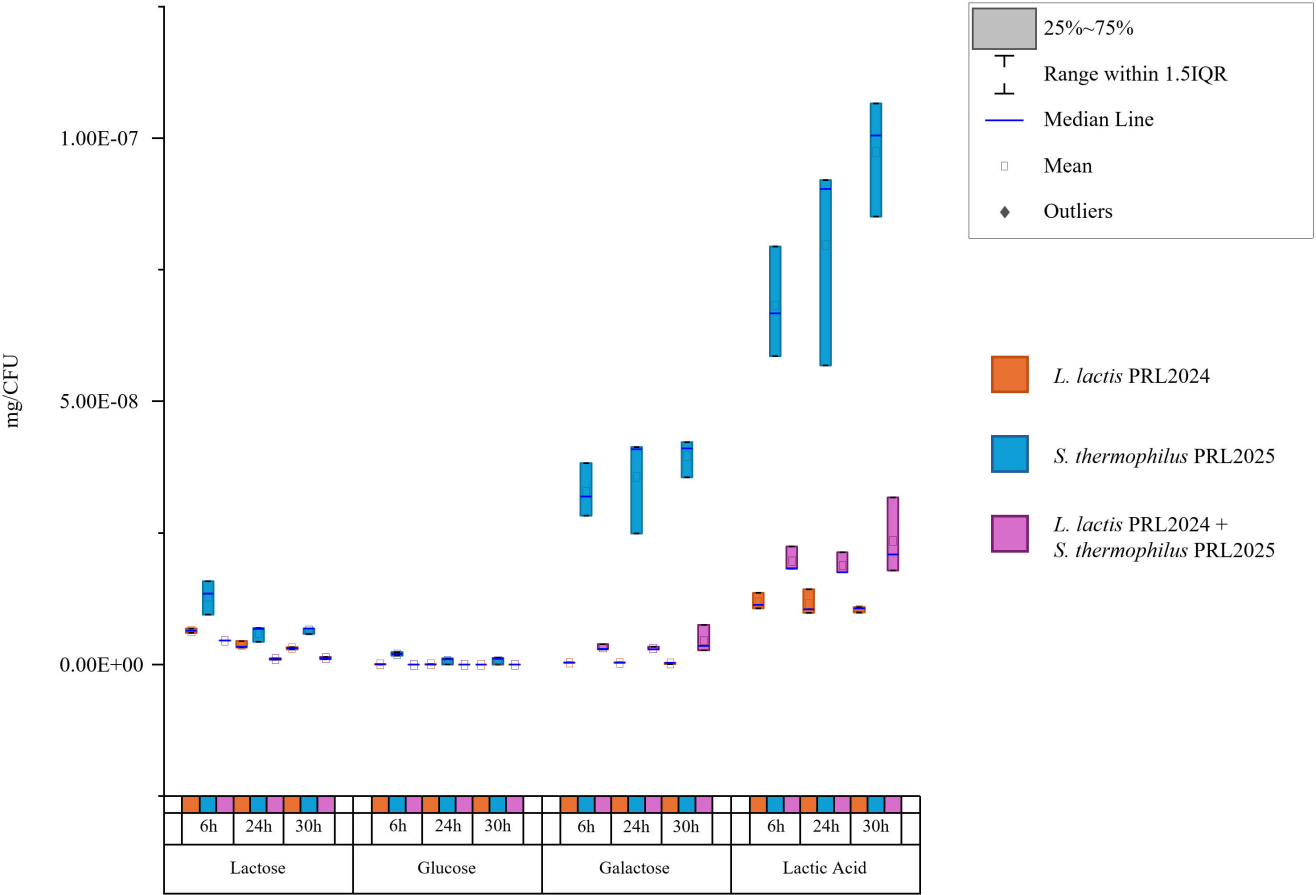
Mono- and bi-association growth performances of PRL2024 and PRL2025 in MRS without glucose + 1% lactose. The chart describes the degradation of lactose in glucose and galactose and the production of lactic acid at three time points (6 h, 24 h, and 30 h). The y-axis represents the normalized metabolite values in mg/CFU of PRL2024 (orange), PRL2025 (blue), and the bi-association (violet).

Then, the consumption of lactose, along with its breakdown into glucose and galactose and the subsequent production of lactic acid, was evaluated for PRL2024, PRL2025, and their association. The analysis revealed that *S. thermophilus* PRL2025 was able to produce a significantly higher amount of lactic acid than *L. lactis* PRL2024 at all three tested time points, i.e., 6 h, 24 h, and 30 h (Fig. 2; Fig. S2). Furthermore, although both strains displayed the same ability to grow on galactose as the unique carbon source (Fig. 1b), their efficiency in metabolizing this monosaccharide was different when it was released from lactose hydrolysis. Although glucose was metabolized under all conditions, *S. thermophilus* PRL2025 left a significant amount of galactose unmetabolized in the medium (Fig. 2). This evidence may be related to the higher production of lactic acid by *S. thermophilus* PRL2025, which may directly interfere with the growth of the strain due to medium acidification. In fact, under this condition, the number of cells of PRL2025 was significantly lower than that of PRL2024 (Table S4), whose growth performance was found to be higher than that of the other *L. lactis* isolated in the frame of this study (Fig. 1c). Nevertheless, when associated in the same medium, galactose was metabolized by PRL2024, and the production of lactic acid was slightly higher than that identified with PRL2024 in mono-association (Fig. 2). These results highlighted a more active lactose metabolism of *L. lactis* PRL2024, which, when associated with *S. thermophilus* PRL2025, limited lactic acid accumulation.

### Molecular interactions of PRL2024 and PRL2025 with species colonizing the human gut microbiota

**Fig 3:**
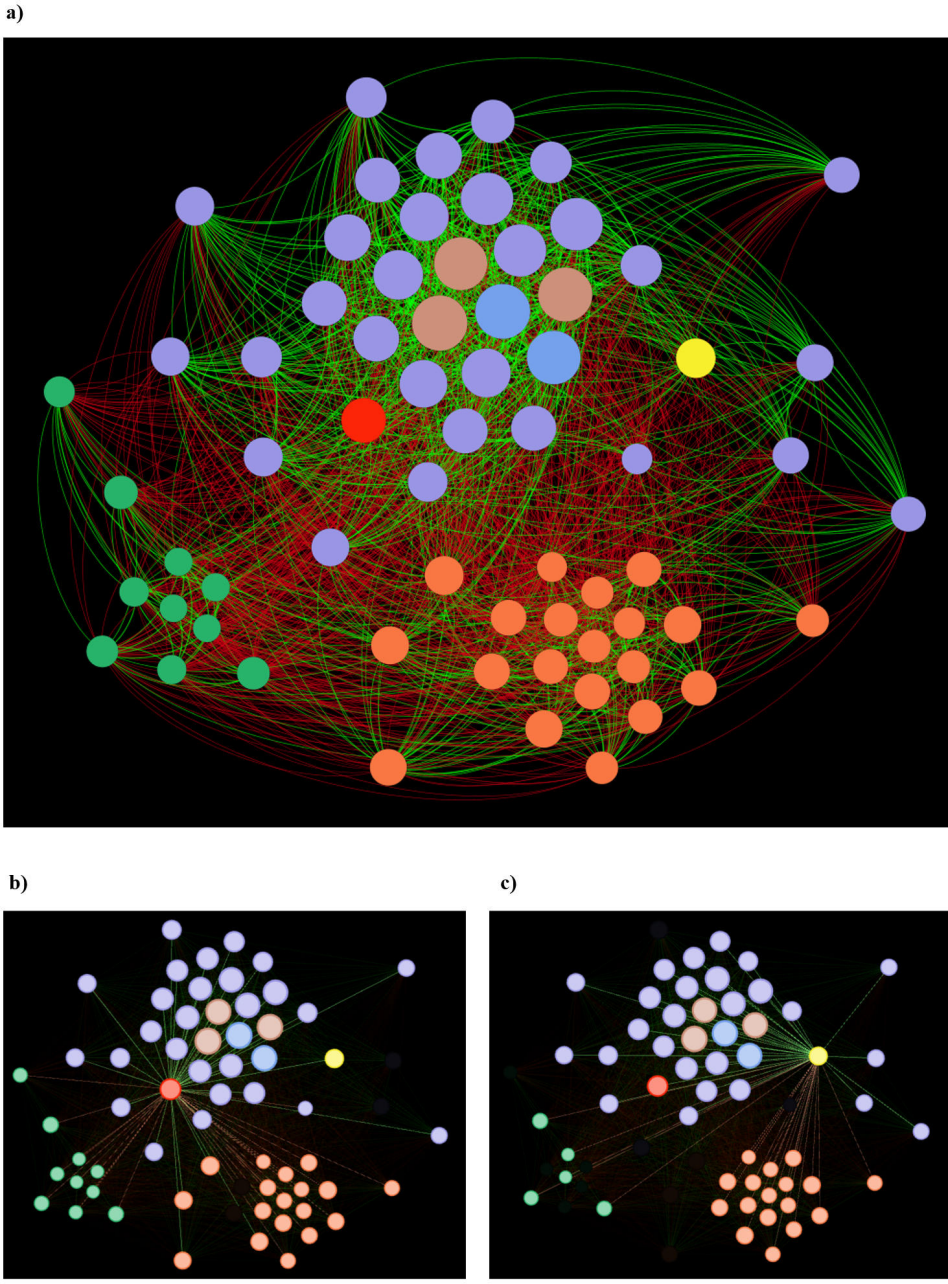
Network of bacterial interactions within the gut microbiota of healthy adults. Panel (**a**) displays a full interaction network with connections among the most significant correlated taxa to *S. thermophilus* and *L. lactis* (Table S5). Panel (**b**) depicts the interaction network, highlighting only the connections involving *S. thermophilus*, while panel (**c**) exhibits the network of *L. lactis*, displaying its specific associations within the gut community. Circles of both species are colored to highlight their interconnection (PRL2025 in yellow and PRL2024 in red) with butyrogenic and propionate-producing bacteria (light red and light blue, respectively). The circle dimension is proportional to the prevalence of the taxa within the human gut, while green and red interconnections represent positive and negative correlation, respectively (Table S1).

To identify which species are more correlated with the presence of *L. lactis* and *S. thermophilus* within the human gut, a covariance analysis involving the distribution of microorganisms in 10,000 healthy adults, as previously reported (23), was explored. The analysis revealed a clear correlation between *S. thermophilus* and butyrogenic bacteria, such as *Anaerostipes hadrus* and *Anaerobutyricum hallii* (36) (Fig. 3; Table S5). Alongside these two bacterial species, *S. thermophilus* correlates with the presence of three different species of the *Blautia* genus, i.e., *Blautia massiliensis*, *Blautia wexlerae*, and *Blautia obeum*. Members of the *Blautia* genus are well-known short-chain fatty acid producers (37), although they were barely associated with the production of butyrate. Nevertheless, the correlation analysis highlighted a strong link between *S. thermophilus* and butyrogenic bacteria. On the other hand, *L. lactis* seems to be associated, in order of co-occurrence significances, with the presence of *Enterococcus faecalis*, *Leuconostoc mesenteroides*, *S. thermophilus*, *Blautia obeum*, and *Dorea formicigenerans* (Fig. 3; Table S5). In contrast to *S. thermophilus*, none of the latter species, which are usually associated with *L. lactis*, are butyrogenic bacteria. Evidence suggests that the higher prevalence of the nomadic *S. thermophilus* within the gut microbiota (Table S1) may be driven by a mutualistic interaction between its lactic acid production and the butyrate biosynthesis of associated gut bacteria.

**Fig 4:**
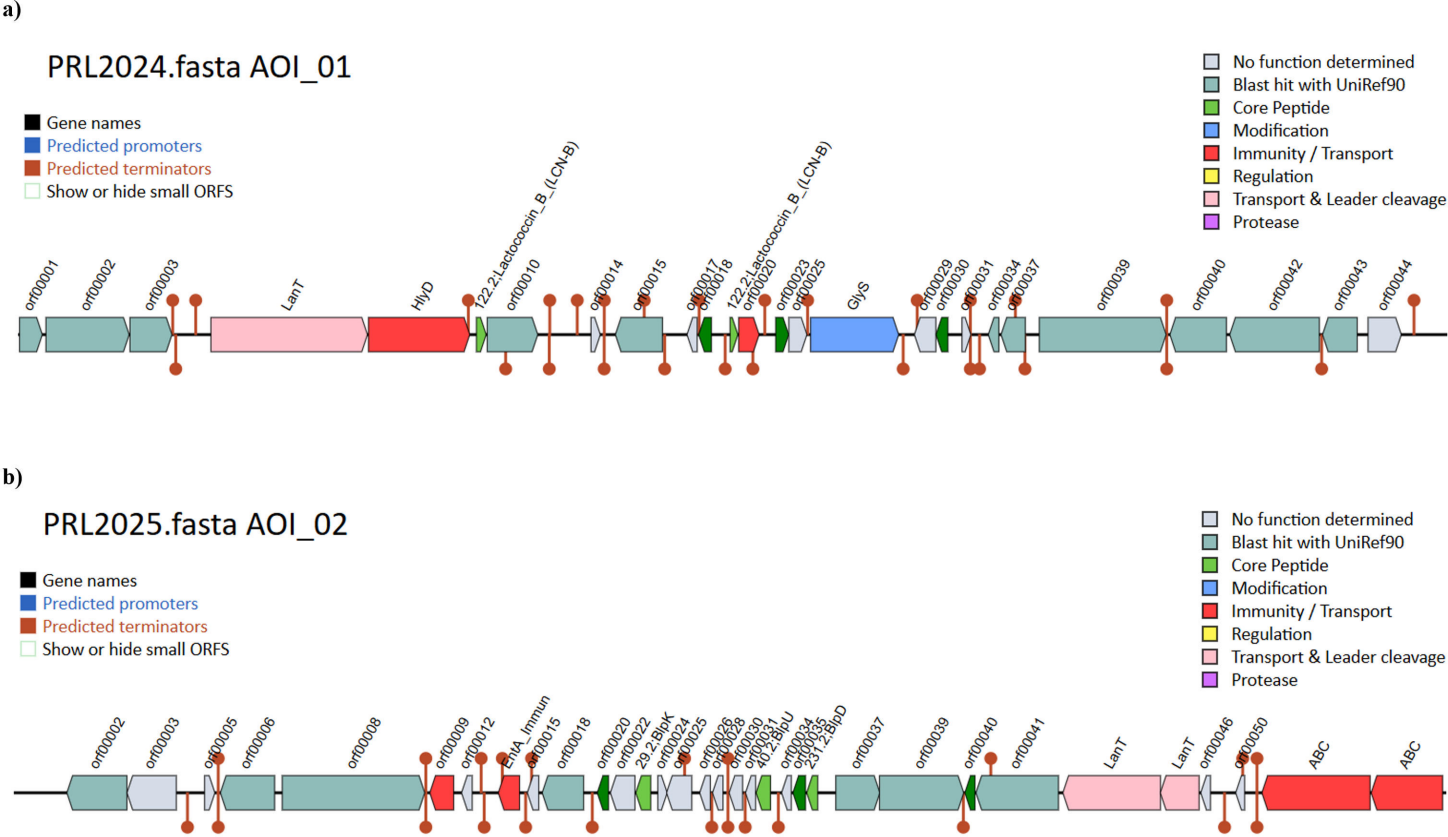
Bacteriocin-encoding loci of PRL2024 and PRL2025. Panels (**a**) and (**b**) report the predicted bacteriocin encoding loci of *L. lactis* PRL2024 and *S. thermophilus* PRL2025, respectively. Genes are shown as colored arrows, such as core peptides of the bacteriocin (light green), genes implicated in the maturation and functionality of the core peptides (dark green), and transport and immunity (red).

To further investigate genomic features owned by the two LAB responsible for the interaction with other microorganisms, a bacteriocin screening encompassing peptides associated with post-translational modifications and transport was performed. PRL2024 was predicted to possess a locus with two bacteriocins (lactococcin-B), three genes involved in their modification, a self-immunity protein, and two dedicated secretion proteins (Fig. 4a). In a similar fashion, PRL2025 showed a locus predicted to encode for three bacteriocin class IIc, as well as three proteins involved in their modification, two self-immunity proteins, and four bacteriocin ABC exporters (Fig. 4b). These findings suggest that PRL2024 and PRL2025 may actively contribute to shaping microbial communities through the production of antimicrobial peptides, which can inhibit competing microorganisms while promoting their own ecological fitness.

### Evaluation of PRL2024 and PRL2025 gastrointestinal resilience and interaction with human epithelial cells

**Fig 5:**
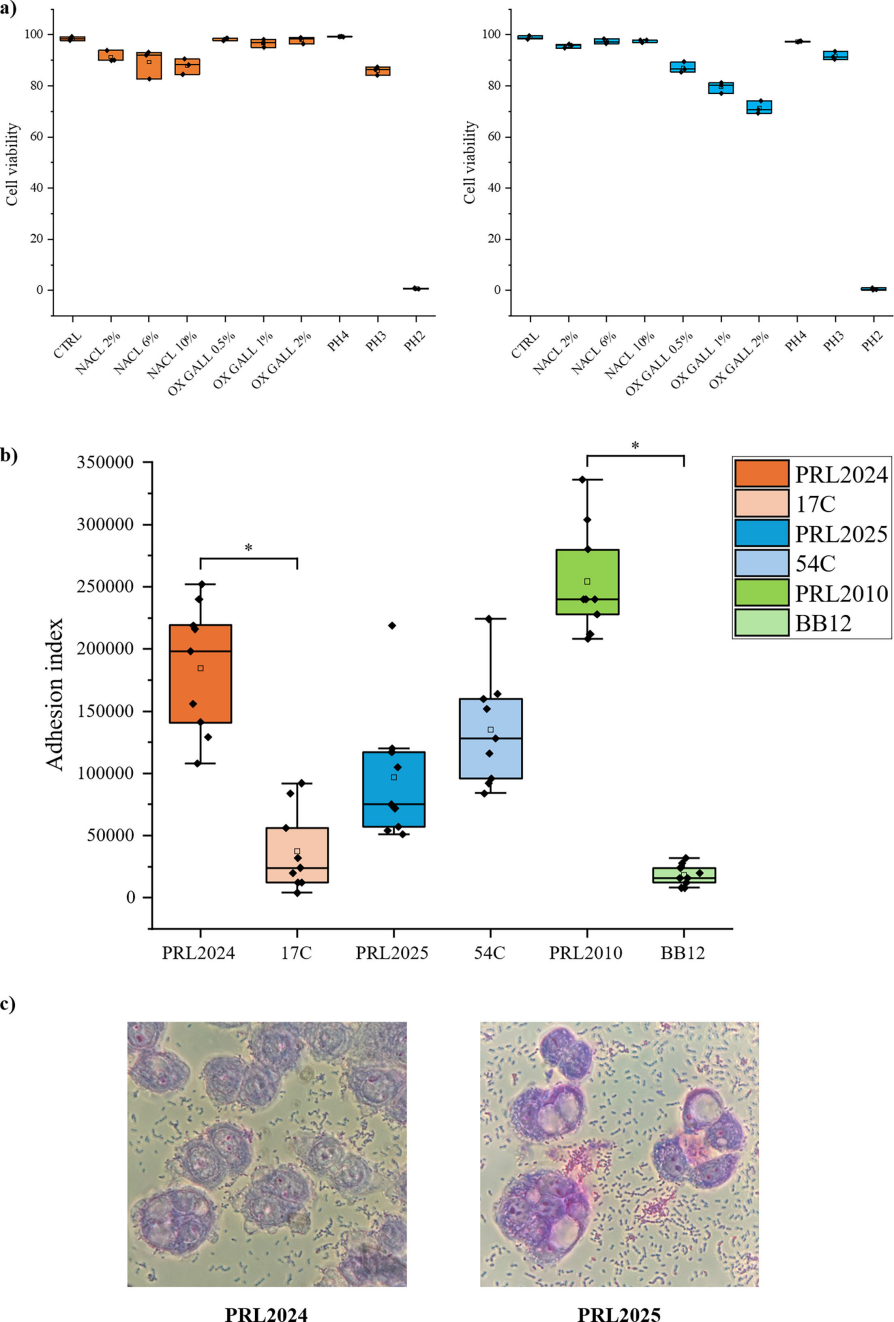
Evaluation of the viability and adhesion performance of PRL2024 and PRL2025 in simulated human gut environments. Panel (**a**) shows the tolerance of the two strains toward human gut challenges. The y-axis represents the percentage of viable cells of PRL2024 (orange) and PRL2025 (blue). Panel (**b**) displays the adhesion index of PRL2024 and PRL2025 cells to HT29-MTX cell monolayers together with two additional LAB strains (17C and 54C) and two bifidobacterial strains (PRL2010 and BB-12). Panel (**c**) exhibits light microscopic images of HT29-MTX monolayer cells as observed with Giemsa staining of PRL2024 and PRL2025 cells.

The ability to withstand the harsh conditions of the GIT, including osmotic stress, bile salts, and acidic environments, is crucial for the successful colonization of enteric bacteria (38). To evaluate the survival ability of PRL2024 and PRL2025, cells were exposed to different chemical agents for 3 hours (39), after which cell viability was monitored through flow cytometry. To assess resistance to osmotic stress, PRL2024 and PRL2025 were exposed to NaCl concentrations of 2%, 6%, and 10% for 3 hours, showing survival rates exceeding 85% and 95%, respectively (Fig. 5a; Table S6). Similarly, tolerance to bile salts was evaluated by exposing the strains to Oxgall (0.5%, 1%, and 2%), where PRL2024 viability was 97% and PRL2025 87% (Fig. 5a; Table S6). Lastly, both strains exhibited high acid resistance, with viabilities of 86% (PRL2024) and 92% (PRL2025) after 2 hours at pH 3, though survival rates dropped significantly at pH 2 (Fig. 5a; Table S6). In contrast, *Bifidobacterium bifidum* PRL2010, which is a well-known commensal of the human gut, showed markedly lower resilience, with survival rates of only 16% at 10% NaCl, 30% at 2% Oxgall, and 16% at pH 3 (40). These findings underscore the exceptional ability of PRL2024 and PRL2025 to withstand key physiological stressors compared to a typical gut commensal. However, the high gastrointestinal resilience of the two LAB reference strains requires further validation through experiments assessing their actual interaction with the human gut. In this context, the well-known probiotic *Bifidobacterium animalis* subsp. *lactis* BB-12 has demonstrated even greater tolerance to acidity and bile salts compared to the two LAB strains (41). Nevertheless, it is also recognized as a transient microorganism that rapidly disappears once administration ceases, without causing lasting changes to the overall composition of the gut microbiota (42, 43).

To obtain a preliminary evaluation of PRL2024 and PRL2025 interaction with the human gut epithelium, we assessed their adhesion to eukaryotic cells (HT29-MTX). Both strains were able to adhere to HT29-MTX cell layers, with adhesion indexes of 184,333 ± 52,007 and 96,667 ± 53,092 for PRL2024 and PRL2025, respectively (Fig. 5b and c). To better evaluate the host-adhesion features of the two reference strains, two additional LAB strains with the lower LAORS score (Table S3) were included in these analyses, as well as two renowned probiotic bacteria, i.e., *B. animalis* subsp. *lactis* BB-12 and *B. bifidum* PRL2010. The latter strains were used to compare adhesion to HT29-MTX cell layers with strains already known to exhibit a marked difference in their ability to interact with human cells (29, 40). The experiment confirmed a significant difference between the two bifidobacterial strains (Mann–Whitney test *P*-value < 0.01; Fig. 5b), highlighting also a significantly larger adhesion index of PRL2024 in comparison to 17C (Mann–Whitney test *P*-value < 0.01; Fig. 5b).

As supported by these findings, *L. lactis* PRL2024 and *S. thermophilus* PRL2025 strains appear to be ecologically adapted to survive and interact with the human gut. Their genetic traits, including an enhanced ability to endure the harsh conditions of the gut environment and to adhere to human epithelial cells, provide valuable insights into their ecological fitness and potential interactions within the gastrointestinal ecosystem.

### LAB gene expression on Caco-2/HT29-MTX monolayers

Several studies reported in scientific literature have explored the transcriptome landscape of *L. lactis* (44, 45) and *S. thermophilus* (46, 47). However, no transcriptomic data have been generated to analyze their gene expression when exposed to human epithelial cells. To date, our understanding of their interaction with human cells is primarily based on their immunomodulatory properties, such as their influence on IFN-γ and TNF-α production (48, 49). Thus, to investigate the genetic mechanisms underlying the interaction between *L. lactis* PRL2024 and *S. thermophilus* PRL2025 with the human host, a transcriptomic analysis was conducted on both strain cells cultured on Caco-2/HT29-MTX cell monolayers.

After quality filtering and ribosomal locus-encompassing read removal, the resulting 33 million reads revealed, when compared with the control condition (LAB grown without Caco-2/HT29-MTX cell monolayers), a statistically significant upregulation of 104 genes in PRL2024 and 102 in PRL2025 (Fig. S3; Table S7). An eggNOG orthology-based prediction revealed differential gene expression patterns between PRL2024 and PRL2025. Specifically, neglecting genes involved in housekeeping functions such as translation and transcription processes, PRL2024 showed an upregulation of 10 genes involved in amino acid metabolism and 9 genes involved in protein homeostasis processes (Table S7). The analysis also highlighted a downregulation of 14 genes involved in carbohydrate metabolism as well as 10 genes related to energy production. Further analysis revealed that PRL2024 did not exhibit clusters of adjacent upregulated genes throughout its genome.

In contrast, PRL2025 exhibited the upregulation of 12 genes involved in cell wall biogenesis and 10 genes predicted to be involved in carbohydrate metabolism and the downregulation of 22 genes involved in amino acid metabolism and 9 genes in inorganic ion metabolism (Table S7). Further analysis of PRL2025 identified five distinct genomic regions characterized by clusters of adjacent upregulated genes upon interaction with Caco-2/HT29-MTX monolayers. Four of these clusters encompassed genes related to essential cellular functions, including genome maintenance, cell wall integrity, cell division, protein synthesis, and RNA maturation (Table S7). The upregulation of these pathways suggests a cellular response aimed at enhancing structural integrity and maintaining active biosynthetic processes during host interaction. More interestingly, the fifth cluster of PRL2025 was predicted to be responsible for the biosynthesis of rhamnose-rich polysaccharides, potentially contributing to EPS formation. These data suggest that PRL2025 may modify its surface architecture to promote adherence to the gastrointestinal, mucus-producing epithelial cells.

### Conclusions

Many fermented foods rely on starter cultures containing LAB, such as yogurt, cheese, and certain cured meats, while other traditional products depend on the natural proliferation of LAB from raw ingredients. As revealed by our human gut microbiome analyses, some of these nomadic LAB are present in the intestinal environment, with *S. thermophilus* being the most prevalent among them. While scientific evidence suggests that food-related bacteria are transient members of the human gut microbiota, a longitudinal meta-analysis performed as part of this study revealed that *S. thermophilus* exhibited stable colonization (prevalence >85% across samples) in seven subjects. These findings support the hypothesis that members of the food microbiota can reach and colonize the human gut via horizontal transmission through food ingestion, reinforcing previous published findings (27).

The PRL2025 reference strain, identified using the LAORS pipeline, was able to grow on a wide range of carbon sources. What makes *S. thermophilus* an interesting resident of the human gut is its correlation with butyrogenic bacteria, such as *Anaerostipes hadrus* and *Anaerobutyricum hallii*, suggesting that its presence may promote the persistence of beneficial bacteria. The other reference strain, *L. lactis* PRL2024, which is also capable of using a wide range of carbon sources, was usually found in the human gut microbiota together with *S. thermophilus*.

Based on these observations, an *in vitro* growth experiment in bi-association revealed that, while *S. thermophilus* PRL2025 produces more lactic acid, it leaves a large amount of galactose unmetabolized, potentially due to medium acidification affecting its growth. In contrast, *L. lactis* PRL2024 shows a more efficient metabolic profile, fully utilizing galactose with modest lactic acid production. Thus, when combined, PRL2024 balances for the PRL2025 metabolism, leading to complete lactose and galactose consumption with a moderate lactic acid accumulation.

The different gene regulation observed in PRL2024 and PRL2025 upon interaction with Caco-2/HT29-MTX monolayers highlights distinct adaptation strategies to the host environment. More specifically, PRL2025 appears to prioritize structural integrity and adhesion, as suggested by the upregulation of its EPS locus, which may enhance its ability to adhere to the human gut epithelium. This is consistent with the well-established role of EPSs as fundamental bacterial structures that mediate interactions with the host (50). Finally, *in vitro* experiments revealed that *L. lactis* PRL2024 and *S. thermophilus* PRL2025 are ecologically adapted to survive and interact within the human gastrointestinal tract. Their genetic traits, including enhanced resilience to the gut environment and strong adhesion to human epithelial cells, underscore their potential as promising candidates for future *in vivo* interaction studies.

## MATERIALS AND METHODS

### Metagenomic data set of the human gut microbiota

The distribution of LAB in the human gut was outlined using data from 10,000 publicly available metagenomes of the human gut, with the shotgun metagenomic data collection previously reported in an earlier study (23). The human gut microbiota profiles were achieved using the METAnnotatorX2 pipeline (51). The latter pipeline was also applied to investigate the distribution of LAB in a subset of longitudinal human gut metagenomes (24).

### LAB strain selection

Complete and partial genome sequences of *L. lactis* and *S. thermophilus* strains were retrieved from the RefSeq NCBI database, representing a collection of publicly available genome sequences of these taxa (23). Genome sequences were validated employing fastANI (average nucleotide identity [ANI] > 94%) (52), CheckM (completeness >95%) (53), and de-replicated using dRep v2.0 (identity >99.8%) (54). Then, the LAORS pipeline was employed to identify reference strains for the *in vitro* analyses, utilizing InStrain (55) to calculate ecological, genomic, and functional scores (23).

### Genome sequencing

Genome sequences of 7 *L. lactis* and 19 *S. thermophilus* strains were determined by GenProbio Srl (Parma, Italy) using a MiSeq platform (Illumina, San Diego, CA) and a NextSeq 2000 platform (Illumina, San Diego, CA). Genome libraries were prepared using an Illumina Nextera XT DNA Library Preparation Kit (Illumina, San Diego, CA). Libraries were quantified using a fluorometric Qubit quantification system (Life Technologies, USA), loaded on a 2200 TapeStation instrument (Agilent Technologies, USA), and normalized to 4 nM. Sequencing was performed using a MiSeq Reagent Kit v3 (600-cycles; Illumina, San Diego, CA), a NextSeq 1000/2000 Reagents Kit (300-cycles; Illumina, San Diego, CA), and a deliberate spiking of 1% PhiX control library. PRL2024 and PRL2025 extracted DNA were also subjected to whole-genome sequencing using the Nanopore DNA sequencing platform according to the supplier’s protocol (Oxford Nanopore, UK).

### Genome assembly

Long reads of PRL204 and PRL2025 were filtered by quality using the Filtlong tool (https://github.com/rrwick/Filtlong), while short reads were filtered through the fastq-mcf script (https://github.com/ExpressionAnalysis/ea-utils) and the BBMap tool (https://github.com/BioInfoTools/BBMap). Filtered fastq ﬁles of Nanopore long reads obtained from genome sequencing efforts were then used as input for genome assembly through CANU v2.2 software (56). The resulting genome sequence has been polished through Polypolish (57) using Illumina paired-end reads. The whole process was managed by the MEGAnnotator2 pipeline (58), as well as short-read assemblies of the other 24 LAB strains (Table 1).

### Comparative genomics profiling

Reference strain proteomes were screened for genes predicted to encode carbohydrate-active enzymes based on sequence similarity to genes classified in the CAZy database (59). Thus, each gene sequence was screened for orthologs through the dbCAN3 pipeline (60) using HMMER v3.3.2 (cutoff e-value of 1 × 10^−15^ and coverage >0.35) against the dbCAN and dbCAN-sub databases and DIAMOND (cutoff *e*-value <1 × 10^−102^) against the CAZy database. Positive results on all three databases were used to predict the glycobiome of each strain. Then, the presence and location of signal peptide cleavage sites in amino acid sequences were identified using the SignalP 4.1 software (61). Furthermore, BAGEL5 software was used to mine bacteriocins in LAB genomes (62) and the eggNOG 5.0 database for systematic analysis of gene functions in the RNAseq experiment (63).

### LAB cultivation conditions

LAB cells were cultivated in M-17 medium (Oxoid, Thermo Scientific, US) supplemented with 1% (wt/vol) lactose (Merck, Germany) and 0.05% (wt/vol) L-cysteine hydrochloride (Merck, Germany) and incubated at 37°C in an anaerobic cabinet (Concept 400, Ruskinn) with an anaerobic atmosphere (2.99% H_2_, 17.01% CO_2_, and 80% N_2_).

### Cultivation conditions of bifidobacteria

*B. animalis* subsp. *lactis* BB-12 and *B. bifidum* PRL2010 were cultivated in MRS medium (Sharlau Chemie, Spain) supplemented with 0.05% (wt/vol) L-cysteine hydrochloride (Merk, Germany) and incubated at 37°C in an anaerobic cabinet (Concept 400, Ruskinn) with an anaerobic atmosphere (2.99% H_2_, 17.01% CO_2_, and 80% N_2_).

### Carbohydrate growth assays

To validate our *in silico* findings, we performed growth assays of PRL2024 and PRL2025 using selected carbon sources. Both strains were cultivated overnight on a semisynthetic M-17 medium supplemented with 1% (wt/vol) lactose (Merck, Germany) and 0.05% (w/vol) L-cysteine hydrochloride at 37°C under anaerobic conditions. Then, cells were diluted in MRS without glucose to obtain an OD600 ~1, and 15 µL of the diluted cells was inoculated in 135 µL of MRS without glucose, supplemented with 1% (wt/vol) of a specific sugar, in a 96-well microtiter plate and incubated in an anaerobic cabinet. Specifically, each carbohydrate was dissolved in MRS without glucose, previously sterilized by autoclaving at 121°C for 15 min. Subsequently, each obtained solution was filter sterilized using a 0.2 µm filter size prior to use. Cell growth was evaluated by monitoring the optical density at 600 nm with the use of a plate reader (Biotek, VT, USA). The plate was read in discontinuous mode, with absorbance readings performed at 3 min intervals for three times after 48 h of growth, and each reading was performed following 30 s of shaking at medium speed. Cultures were grown in triplicate, and the resulting growth data were expressed as the average of these replicates. Carbohydrates tested in this study were purchased from Merck (Germany) and include arabinose, fructose, glucose, mannitol, rhamnose, ribose, sorbitol, xylose, lactose, maltose, melibiose, sucrose, trehalose, turanose, soluble starch from potato, raffinose, lactulose, cellobiose, fucose, galactose, mannose, N-acetyl-D-glucosamine, N-acetyl-D-galactosamine, inulin, amylopectin, pullulan, maltotriose, maltodextrin, glycogen, fructooligosaccharide, arabinogalactan, xylan, and mucin from porcine stomach.

### Sugars and lactic acid quantification

The concentration of lactose, galactose, glucose, and lactic acid was quantified by means of high-performance liquid chromatography (HPLC) (1200 System, Agilent Technologies, Waldbronn, Germany), equipped with an Aminex HPX-87 H ion exchange column (Bio-Rad, Hercules, CA, USA) and a refractive index detector. Isocratic elution was carried out at 60°C with 0.6 mL min^−1^ of 5 mM H_2_SO_4_. The samples were clarified by centrifugation (10,000 × *g* for 5 min) and filtration at 0.22 µm before injection. The experiments were carried out in triplicate.

### Assessment of PRL2024 and PRL2025 tolerance to various stress conditions

To assess the pH tolerance of PRL2024 and PRL2025, microbial cells were grown in 10 mL of M-17 broth at 37°C under anaerobic conditions until reaching a final concentration of 10^8^ cells/mL. Then, cells were centrifuged at 930 × *g* for 8 min, washed with phosphate-buffered saline (PBS, pH 6.5), and resuspended in 10 mL of M-17 broth, adjusted to pH 2.0, pH 3.0, or pH 4.0 with the addition of HCl. Finally, LAB cells were incubated under anaerobic conditions at 37°C for 2 h, as previously described (39). The same procedure was performed to assess the ability of LAB to tolerate different NaCl (2%, 6%, and 10%) or bile salts (Merck, Germany; 0.5%, 1%, and 2%) concentrations with an exposure of 3 h to these stressful conditions, as previously reported (39). Experiments were carried out in triplicate, and a control sample was obtained by inoculating LAB cells in M-17 broth. After exposure to acidic environments, different bile salt concentrations, or varying levels of NaCl, a 10-fold serial dilution in PBS was obtained from each tested condition. The diluted cells were then analyzed for viability using flow cytometry with the fluorescent dyes SYTO9 (3.34 mM) and PI (20 mM) of the LIVE/DEAD BacLight Bacterial Viability kit (ThermoFisher Scientific, USA), following the manufacturer’s protocol (Manual of the LIVE/DEAD BacLight Bacterial Viability and counting kit, ThermoFisher Scientific, USA). Briefly, two aliquots of 1 mL of bacterial cell dilution (1:100) were harvested by centrifugation at 3,000 rpm for 6 min and washed with PBS. Subsequently, one of the two aliquots of bacterial suspension was exposed to 70% isopropyl alcohol and kept on ice for 1 h to permeabilize cell membranes and induce cell death, while the other 1 mL aliquot was maintained in PBS to preserve cell viability. Then, 1.5 µL of a specific dye was added to samples for the single staining assay, while for the double staining assay, 1.5 µL of both dyes was added to samples. Once stained, samples were incubated in the dark for 15 min at room temperature. While single-stained controls were used for instrument parameter adjustment, non-stained cells were used as background control. Cell viability assay was performed with the Attune NxT flow cytometer (ThermoFisher Scientific, USA), and all data were analyzed with the Attune NxT flow cytometer software.

### Experimental treatments on human cell monolayers

Human colorectal adenocarcinoma-derived Caco-2 cells, commonly employed for *in vitro* studies of intestinal cell function and differentiation because of their ability to express differentiation features typical of mature intestinal cells (64), were obtained from ATCC. HT29-MTX cells, obtained from Prof. Baldi, University of Milan, are human colon carcinoma-derived, mucin-secreting goblet cells widely exploited to study the influence of mucus on different biological endpoints *in vitro*. Caco-2 cells were cultured in Minimum Essential medium, while HT29-MTX cells were maintained in high glucose (4.5 g/L) Dulbecco’s Modified Eagle’s medium (DMEM). The final concentration of glutamine was 2 mM for Caco-2 cells, whereas the medium of HT29-MTX cells was supplemented with glutamine (4 mM) and sodium pyruvate (10 mM). All media were supplemented with antibiotics (100 µg/mL streptomycin and 100 U/mL penicillin) and 10% fetal bovine serum (FBS; Gibco, Thermo Fisher, Milan, Italy). Cells were maintained in 5% CO_2_ at 37°C and were passaged three times per week. For the experiments, a mixed suspension of Caco-2 and HT29-MTX cells (7:3) was seeded into cell culture inserts (Becton, Dickinson and Company, Franklin Lakes, NJ, United States) at a density of 10^5^ cells/cm^2^ as previously described (65). The cells were cultured for 21 days with medium replacement every 3 days until a tight monolayer was formed (TEER > 600 Ω·cm^2^).

### Adhesion of *L. lactis* PRL2024 and *S. thermophilus* PRL2025 to HT29-MTX cells

Bacterial adhesion to HT29-MTX cells was assessed following the protocol described by Serafini et al. (40). Briefly, human colorectal adenocarcinoma HT29-MTX cells were cultured in DMEM supplemented with 10% FBS, 2 mM glutamine, 100 µg/mL streptomycin, and 100 U/mL penicillin and maintained in standard culture conditions. For the experiments, HT29-MTX cells were seeded on microscopy cover glasses previously settled into 10 cm^2^ petri dishes. Confluent cells were carefully washed twice with PBS before bacterial cells were added. *L. lactis* PRL2024 and *S. thermophilus* PRL2025 were grown as previously described, until a concentration of 5 × 10^7^ CFU mL^−1^ was reached. The strains were then centrifuged at 3,000  rpm for 8 min, resuspended in PBS (pH 7.3), and incubated with monolayers of HT29-MTX cells. After 1 h incubation at 37°C, cultures were washed twice with 2 mL of PBS to remove unbound bacteria. The cells were then fixed with 1 mL of methanol, incubated for 8 min at room temperature, stained with 1.5 mL of Giemsa stain solution (1:20; Sigma-Aldrich, Milan, Italy), and left in the dark for 30 min at room temperature. After two washes with 2 mL of PBS, the cover glasses were removed from the petri plate, mounted on a glass slide, and examined using a phase-contrast microscope Zeiss Axiovert 200 (objective, 100×/1.4 oil). Adherent bacteria in 20 randomly selected microscopic fields were counted and averaged. The proportion of bacterial cells that remained attached to the HT29-MTX monolayer was determined to reflect the extent of specific host-microbe interaction. The adhesion index represents the average number of bacterial cells attached to 100 HT29-MTX cells (66–68). All assays were performed at least in triplicate.

### LAB RNA extraction

Total RNA from bacterial cells was isolated using a previously described method (69, 70). Briefly, LAB cell pellets were resuspended in 1 mL of QIAzol lysis reagent (Qiagen, Germany) in a sterile tube containing glass beads. Cells were lysed by alternating 2 min of stirring the mix on a bead beater with 2 min of static cooling on ice. This process was repeated three times. Lysed cells were centrifuged at 12,000 rpm for 15 min, and the upper phase was recovered. Bacterial RNA was subsequently purified using the RNeasy Mini Kit (Qiagen, Germany) following the manufacturer’s instructions. Then, RNA concentration and purity were evaluated using a spectrophotometer (Eppendorf, Germany).

### mRNA sequencing analysis

Total bacterial RNA (from 100 ng to 1 µg) was treated with the Illumina Stranded Total RNA Prep, Ligation with Ribo-Zero Plus, Ribo-Zero Plus Microbiome kit to remove rRNA, following the manufacturer’s instructions (Illumina, San Diego, USA). The efficiency of rRNA depletion was assessed using a 2200 TapeStation (Agilent Technologies, USA). Then, a whole transcriptome library for prokaryotic RNA was constructed using a NextSeq 1000/2000 Reagents preparation kit (Illumina, San Diego, USA). The obtained reads were filtered to remove low-quality reads using the fastq-mcf tool (minimum mean quality 20 and minimum length 100 bp) as well as any remaining ribosomal locus-encompassing reads (51). Retained reads were then aligned to the complete, PRL2024, and PRL2025 genome sequence through Bowtie2 software (71). Then, quantification of reads mapped to individual transcripts was achieved through the htseq-counts script of HTSeq software in “union” mode (72). Raw counts were then normalized using the trimmed mean of *M*-values method implemented in the EdgeR package (version 3.6.1) (73), and Log2 fold change (logFC) was used to evaluate the differences in gene expression of PRL2024 and PRL2025 cultivated alone (reference condition) and in bi- or multi-associations (test conditions). The EdgeR package was also used to identify differentially expressed genes at a false discovery rate (FDR) of 5% and a minimal logFC of 1.

### Statistical analysis

Similarities between samples (beta-diversity) were calculated by the Bray-Curtis dissimilarity matrix based on species abundance using the “vegdist” function on RStudio (http://www.rstudio.com/). Correlation analysis between bacterial species of all samples was performed through Spearman’s rank correlation coefficient using “rcorr” function (https://CRAN.R-project.org/package=Hmisc), and only results that were significantly different from a statistical perspective were retained. The FDR correction was applied to all statistical analyses based on Benjamini and Hochberg Correction (74), using RStudio through “p.adjust” function.

## Data Availability

Genome sequences of LAB have been deposited in the GenBank database under the NCBI BioProject PRJNA1248751 and will become publicly available once accession formalization resumes. Raw sequences of transcriptomics experiments can be accessed through the SRA study PRJNA1248712.

## SUPPLEMENTAL MATERIAL

Figure S1 aem.01547-25-s0001.tif Distribution of *S. thermophilus* and *L. lactis* in a longitudinal screening of 35 human gut metagenomes at baseline (pre-treatment).

Figure S2 aem.01547-25-s0002.tif Profiling of the growth media used for the identification of lactose degradation and lactic acid production of PRL2024 and PRL2025.

Figure S3 aem.01547-25-s0003.tif Volcano plot of differential gene expression in response to contact with HT29-MTX cells.

Supplemental legends aem.01547-25-s0004.docx Legends for Fig. S1 to S3.

Supplemental tables aem.01547-25-s0005.xlsx Tables S1 to S7.

ASM does not own the copyrights to Supplemental Material that may be linked to, or accessed through, an article. The authors have granted ASM a non-exclusive, world-wide license to publish the Supplemental Material files. Please contact the corresponding author directly for reuse.
