# Supplementary figures and images for: Host interactions of Lactococcus lactis and Streptococcus thermophilus support their adaptation to the human gut microbiota
Source: Appl Environ Microbiol. 2025 Nov 4;91(12):e01547-25. doi: 10.1128/aem.01547-25 (PMC12724283; doi:10.1128/aem.01547-25)

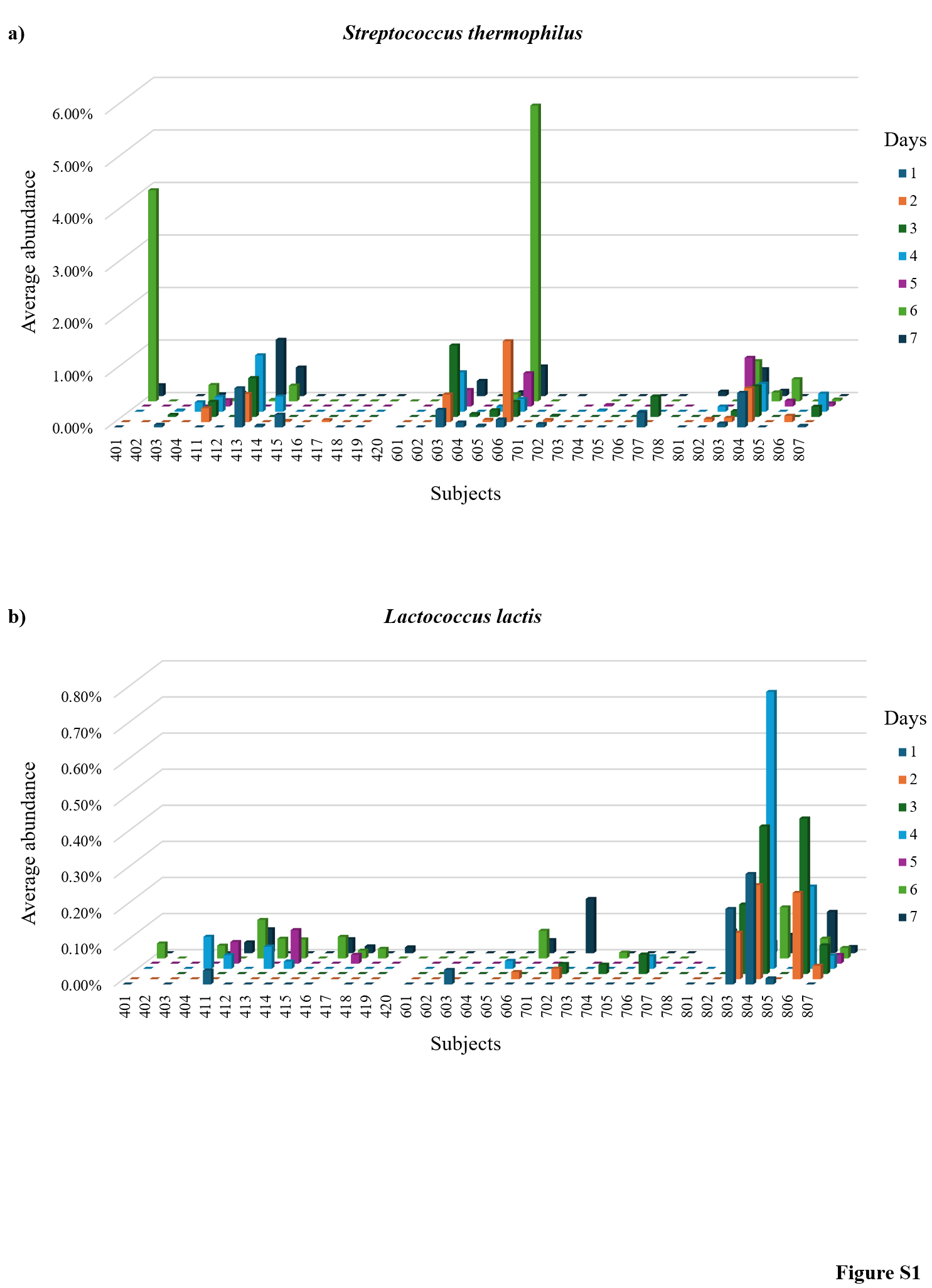

Supplement: Figure S1 — Distribution of S. thermophilus and L. lactis in a longitudinal screening of 35 human gut metagenomes at baseline (pre-treatment). [file aem.01547-25-s0001.tif]

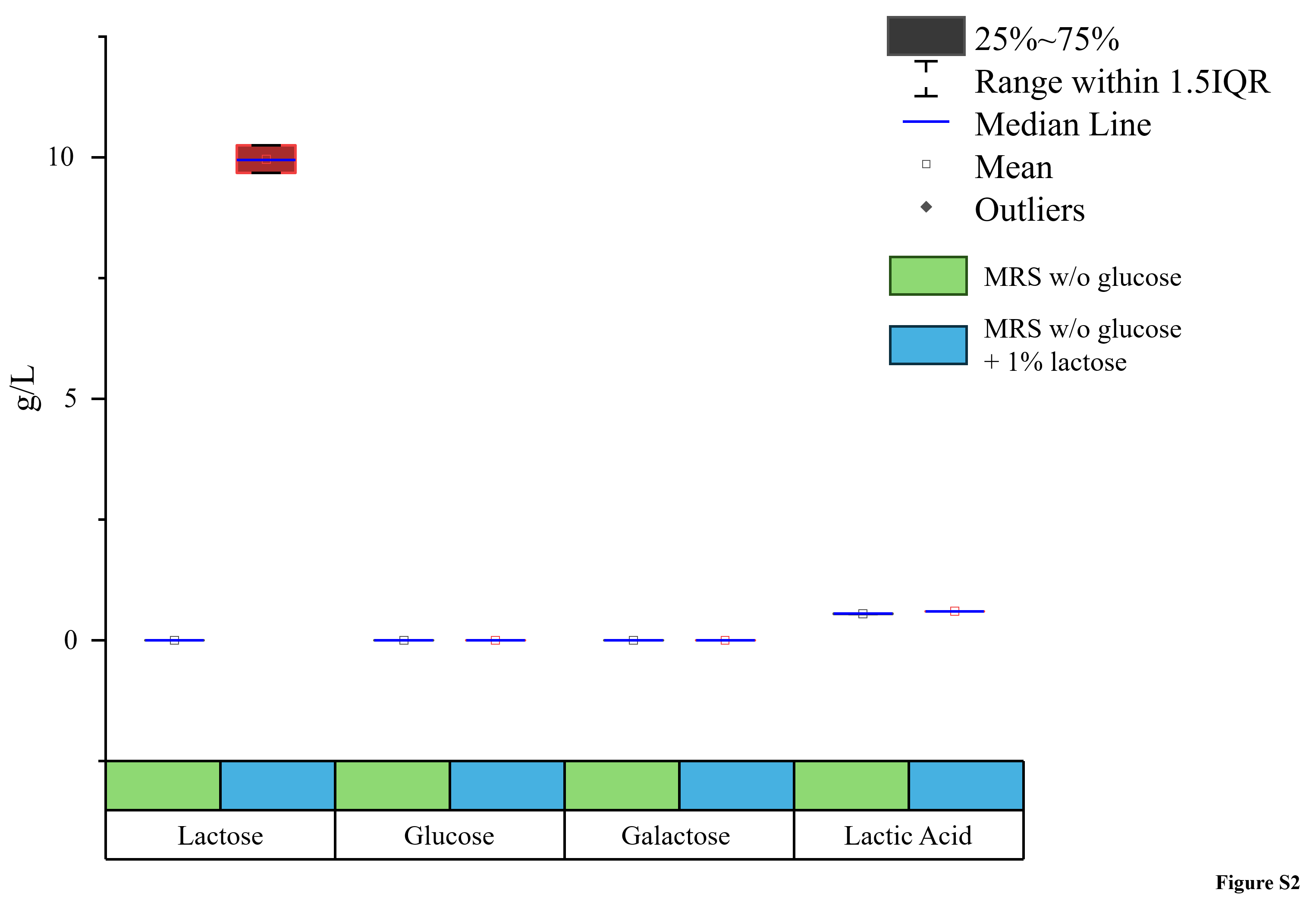

Supplement: Figure S2 — Profiling of the growth media used for the identification of lactose degradation and lactic acid production of PRL2024 and PRL2025. [file aem.01547-25-s0002.tif]

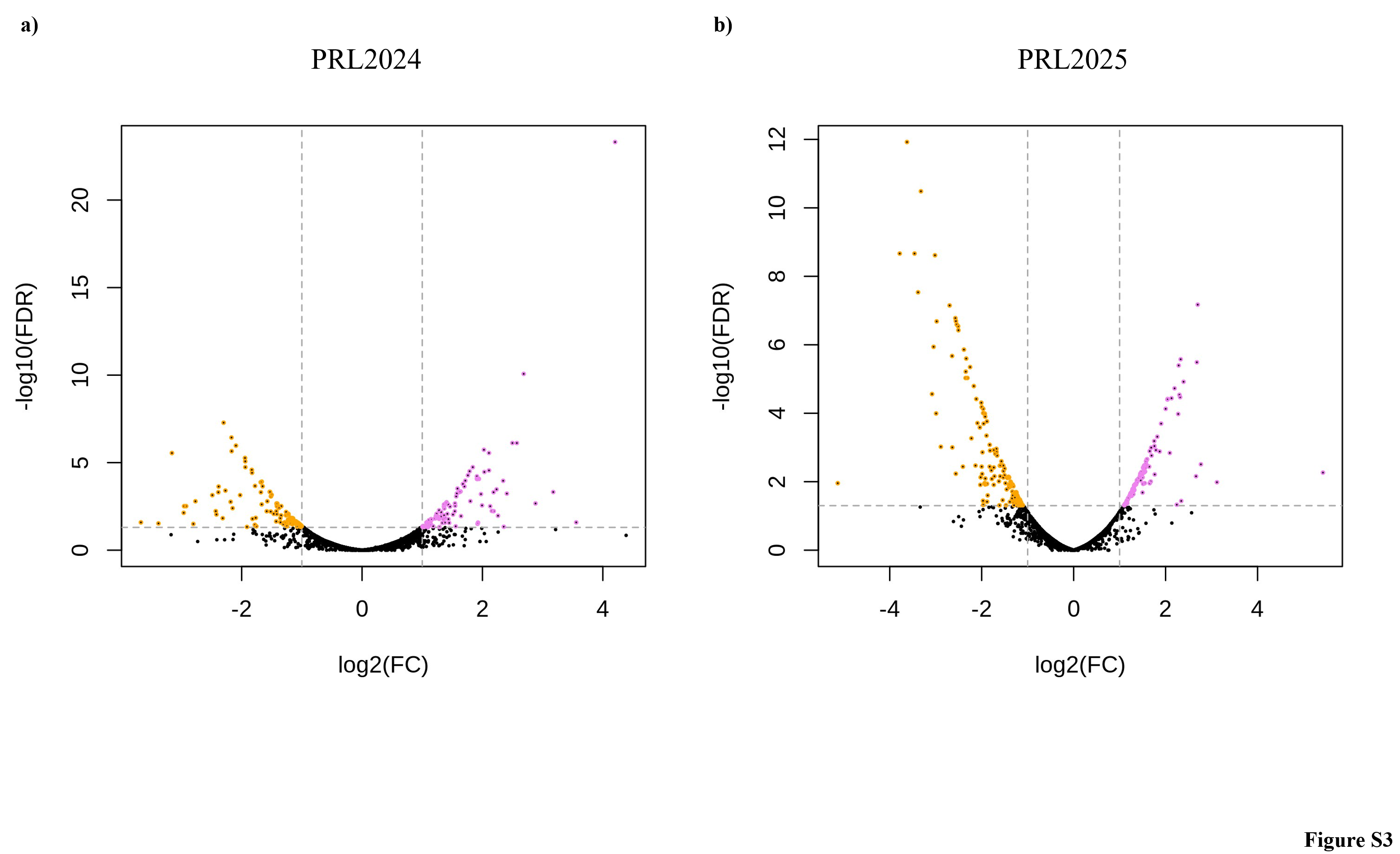

Supplement: Figure S3 — Volcano plot of differential gene expression in response to contact with HT29-MTX cells. [file aem.01547-25-s0003.tif]
