# Supplementary material for: Host interactions of Lactococcus lactis and Streptococcus thermophilus support their adaptation to the human gut microbiota
Source: Appl Environ Microbiol. 2025 Nov 4;91(12):e01547-25. doi: 10.1128/aem.01547-25 (PMC12724283; doi:10.1128/aem.01547-25)
Supplement: Supplemental legends — Legends for Fig. S1 to S3. [file aem.01547-25-s0004.docx]

***Lactococcus lactis* and *Streptococcus thermophilus* as main lactic acid bacteria inhabiting the human gut microbiota**

Running title: Lactic acid bacteria and human gut microbiota

Keywords: lactic acid bacteria, genomics, metagenomics, microbiome, host-microbe interaction

Gabriele Andrea Lugli^1,2^, Chiara Argentini^1^, Chiara Tarracchini^1^, Giulia Longhi^1^, Leonardo Mancabelli^2,3^, Massimiliano G. Bianchi^2,4^, Giuseppe Taurino^2,4^, Alberto Amaretti^5,6^, Francesco Candeliere^5^, Ovidio Bussolati^2,4^, Christian Milani^1,2^, Francesca Turroni^1,2^, Marco Ventura^1,2^

^1^Laboratory of Probiogenomics, Department of Chemistry, Life Sciences, and Environmental Sustainability, University of Parma, Parma, Italy; ^2^Microbiome Research Hub, University of Parma, Parma, Italy; ^3^Department of Medicine and Surgery, University of Parma, Parma, Italy, ^4^Laboratory of General Pathology, Department of Medicine and Surgery, University of Parma, Parma, Italy, ^5^Department of Life Sciences, University of Modena and Reggio Emilia, Modena, Italy, ^6^Biogest-Siteia, University of Modena and Reggio Emilia, Reggio Emilia, Italy.

*Correspondence. Mailing address for Marco Ventura, Laboratory of Probiogenomics, Department of Chemistry, Life Sciences, and Environmental Sustainability, University of Parma, Parco Area delle Scienze 11a, 43124 Parma, Italy. Phone: ++39-521-905666. Fax: ++39-521-905604. E-mail: marco.ventura@unipr.it

**Figure S1.** Distribution of *S. thermophilus* and *L. lactis* in a longitudinal screening of 35 human gut metagenomes at baseline (pre-treatment). Panel a shows the distribution of *S. thermophilus* across participants over seven days, while panel b depicts the corresponding data for *L. lactis*.

**Figure S2.** Profiling of the growth media used for the identification of lactose degradation and lactic acid production of PRL2024 and PRL2025. The y-axis represents the metabolite values in g/L of MRS without glucose (green) and MRS without glucose + 1% lactose (blue).

**Figure S3.** Volcano plot of differential gene expression in response to contact with HT29-MTX cells. Panel a) displays the gene expression of PRL2024, while panel b) shows data of PRL2025. Each dot represents a gene, plotted according to its log2 fold change (x-axis) and –log10 adjusted p-value (y-axis). Orange dots indicate significantly downregulated genes upon contact with human cells (log2 fold change < 0, adjusted p-value < 0.05), while pink dots represent significantly upregulated genes (log2 fold change > 0, adjusted p-value < 0.05). Black dots denote genes without significant changes in expression.
